# Three-dimensional printing in modelling mitral valve interventions

**DOI:** 10.1186/s44156-023-00024-x

**Published:** 2023-08-02

**Authors:** Apurva H. Bharucha, John Moore, Patrick Carnahan, Philip MacCarthy, Mark J. Monaghan, Max Baghai, Ranjit Deshpande, Jonathan Byrne, Rafal Dworakowski, Mehdi Eskandari

**Affiliations:** 1 The Cardiac Care Group, King’s College Hospital, London, SE5 9RS UK; 2 Robarts Research Institute, Western University, London, ON Canada

**Keywords:** Mitral intervention, 3D printing, Future technologies, Personalised care

## Abstract

Mitral interventions remain technically challenging owing to the anatomical complexity and heterogeneity of mitral pathologies. As such, multi-disciplinary pre-procedural planning assisted by advanced cardiac imaging is pivotal to successful outcomes. Modern imaging techniques offer accurate 3D renderings of cardiac anatomy; however, users are required to derive a spatial understanding of complex mitral pathologies from a 2D projection thus generating an ‘imaging gap’ which limits procedural planning. Physical mitral modelling using 3D printing has the potential to bridge this gap and is increasingly being employed in conjunction with other transformative technologies to assess feasibility of intervention, direct prosthesis choice and avoid complications. Such platforms have also shown value in training and patient education. Despite important limitations, the pace of innovation and synergistic integration with other technologies is likely to ensure that 3D printing assumes a central role in the journey towards delivering personalised care for patients undergoing mitral valve interventions.

## Highlights

- Heart Team based pre-procedural planning using advanced cardiac imaging is crucial to successful mitral interventions.
- Cardiac imaging requires users to spatially appreciate structures on 2D projections generating an ‘imaging gap’. 3D printing can surmount the imaging gap to enhance procedural planning and offer personalised care.
- Integration of 3D printing technologies with other transformative technologies and large-scale clinical trial data may facilitate widespread clinical translation.

## Introduction

Mitral regurgitation (MR) is the commonest heart valve lesion in the developed world and is associated with considerable morbidity and mortality [1, 2]. Surgical mitral valve intervention (repair or replacement) is the standard of care for severe MR; however, these interventions are sometimes precluded on grounds of unacceptable surgical risk owing to an aging and increasingly comorbid population [3–5].

Transcatheter mitral valve interventions have shown promise as alternative interventions to mitigate the burden of MR in those deemed unsuitable for conventional surgery. Indeed, transcatheter mitral valve repair (TMVr) is now an established treatment strategy with over 100,000 procedures performed globally. Meanwhile, transcatheter mitral valve replacement (TMVR) may offer a viable alternative for those unsuitable for TMVr and in patients with failed surgical bioprostheses, severe mitral annular calcification (MAC) and unsuccessful surgical mitral repair [6].

Despite significant innovation and evolution, surgical and transcatheter mitral valve interventions face considerable technical challenges arising from the anatomical complexities of the mitral valve apparatus and its dynamic interaction with adjacent structures, as well as the heterogeneity within mitral pathologies. Indeed, the saddle shaped mitral annulus has variable dimensions dependent on haemodynamic status and left ventricular (LV) cavity dimensions rendering device sizing challenging. The complex sub-valvular apparatus may hinder device manoeuvrability and risks entrapment. The intimate anatomical relationship between the left ventricular outflow tract (LVOT) and the mitral valve introduces the catastrophic risk of LVOT obstruction in TMVR [7–9]. Meanwhile, edge-to-edge TMVr is limited by anatomical challenges to successful leaflet grasping and device deployment owing to factors such as leaflet length, calcification, myxomatous disease, clefts and large coaptation defects. TMVr also carries the risk of iatrogenic mitral stenosis which has adverse prognostic implications and may preclude further invasive intervention [10, 11]. More broadly, conventional open-heart surgery offers the opportunity to directly visualise and handle anatomical structures intra-operatively thus facilitating patient-specific procedural adjustment at the point of intervention whereas this is precluded in transcatheter interventions.

Heart Team-based pre-procedural planning using advanced multi-modality imaging is crucial for achieving optimal procedural outcomes, especially in transcatheter mitral interventions due to their inherent complexities and challenges. Although advances in cardiac imaging (notably in multi-detector cardiac CT (MDCT) and three-dimensional (3D) transoesophageal echocardiography (TOE)) now enable rendering of highly accurate 3D images of cardiac structures, there are important limitations arising from the lack of dynamic feedback and tactile interaction. Users are also required to spatially appreciate complex 3D anatomical structures on a two-dimensional screen. As such, there is a growing recognition of an ‘imaging gap’ within structural heart interventions which hamstrings effective procedural planning [12, 13].

The advent and rapid evolution of ‘transformative technologies’ in medicine- namely 3D printing, computer-aided modelling (CAD), computational modelling, artificial intelligence (AI) and extended realities- have the potential to bridge the imaging void gap in structural heart interventions. They may herald a paradigm shift in procedural planning by facilitating patient-specific modelling of mitral pathologies and guiding interventional strategy thus potentially improving procedural outcomes. The integration and clinical translation of such technologies may hold considerable value for future training, evaluation of novel mitral technologies and facilitate the delivery of personalised cardiac care [12, 14].

This work provides an overview of 3D printing technologies and workflows followed by an appraisal of the current status and future clinical translation of this technology in modelling mitral interventions.

## Fundamental concepts in three-dimensional printing

**Fig. 1:**
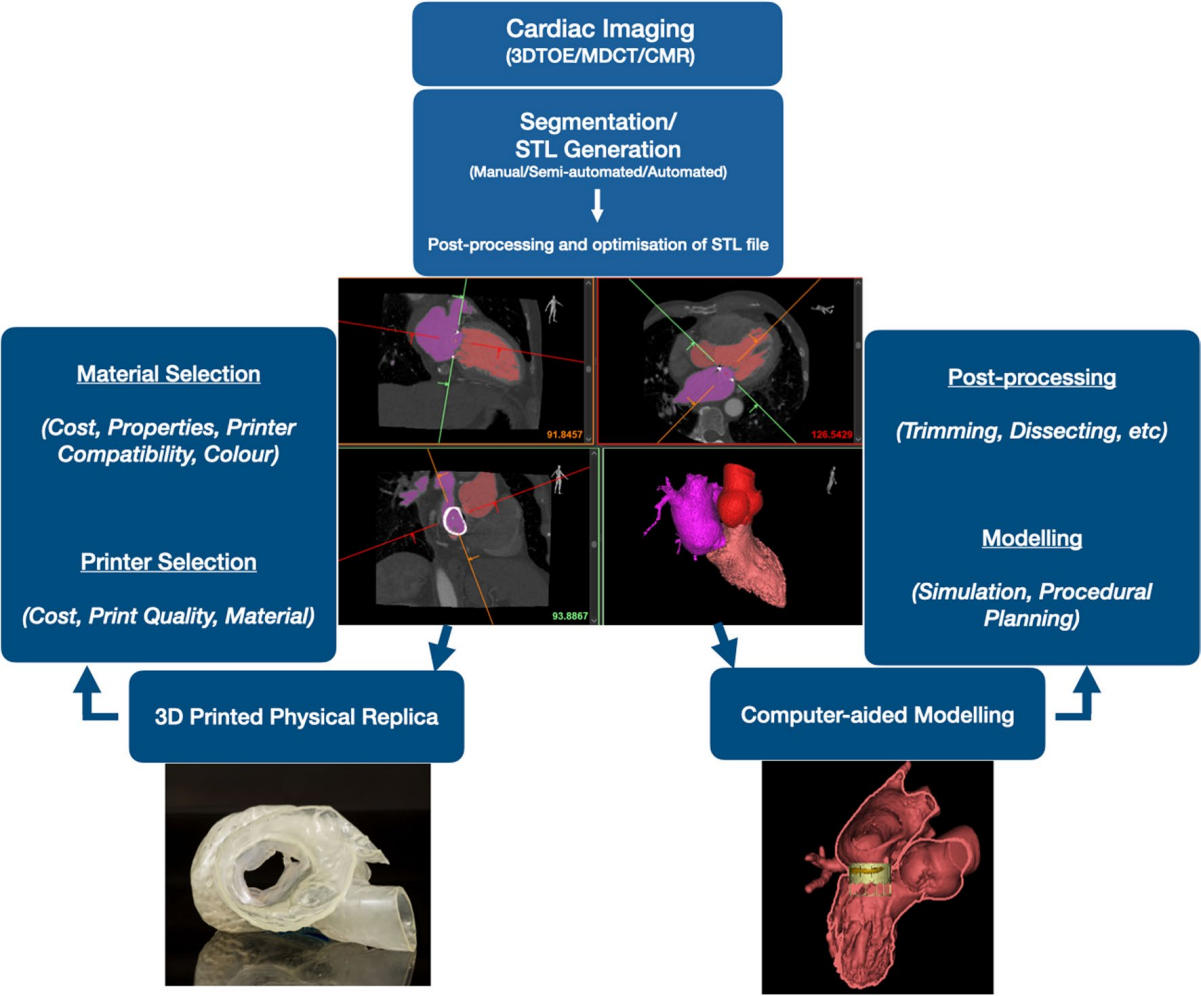
3D printing workflow. 3D printing involves segmentation of a 3D imaging dataset by assigning grey pixels to the anatomical area of interest which is then converted to a file format recognised by a 3D printer**.** (Most commonly Standard Tessellation Language, STL format). The STL file can be either used for printing out a physical replica or incorporated into virtual platforms for computer-aided design and modelling. *3DTOE* Three-Dimensional Echocardiography, *MDCT* Multi Detector Computational Tomography, *CMR* Cardiac Magnetic Resonance

Three-dimensional printing transforms digital objects into physical 3D replicas through multi-layered material deposition over a digitally defined geometry. Figure 1 illustrates the standard workflow involved in cardiovascular 3D printing which comprises broadly of deriving a virtual 3D model of the relevant structure from cardiac imaging (a common step to both 3D printing and computer aided modelling) followed by the manufacture of a physical replica [15, 16].

### Virtualisation of medical imaging

#### Acquisition of cardiac imaging

A volumetric imaging dataset in DICOM (Digital Imaging and Communication in Medicine) format is a prerequisite for segmentation. As such, relevant imaging modalities include ECG gated MDCT, 3D echocardiography and Cardiac MRI (CMR) [17].

MDCT is now the modality of choice for modelling mitral interventions particularly in the transcatheter domain. Indeed, advances in MDCT technology have enabled sub-millimetre spatial resolution (0.3–0.625 mm) which can be obtained with rapid image acquisition speeds. Meanwhile, progress in reconstruction algorithms enable highly accurate virtual 3D renderings of cardiac structures. The use of iodinated contrast agents in MDCT still requires caution in those with chronic renal impairment and CT based modelling involves image acquisition throughout the cardiac cycle thereby conferring additional exposure to ionising radiation. MDCT acquisition can also be limited by arrhythmia and low cardiac output states [18]. MDCT enables multi-dimensional manipulation of reconstructed images and thin slice reconstruction, allowing precise visualisation of fine anatomical details however, this incurs the cost of amplifying imaging artefacts. Accentuated imaging artefact may require substantial post-processing and typically laborious manual segmentation risking the addition of user error in the segmentation process. Alternatively, image reconstruction with thicker slices sacrifices anatomical detail but mitigates the impact of artefact and may be amenable to more automated segmentation and post processing. Cardiac models have been shown to demonstrate sufficient anatomical accuracy with 0.5 mm reconstructions [19]. Cardiac CT offers both volume and surface rendering of the acquired dataset. The latter sharply defines objects in binary fashion as present or absent, facilitating conversion to a mesh required for 3D printing. Volume rendered images are more visually attractive but derived through applying transparency to objects within an acquired dataset and therefore cannot be used for 3D printing [20].

3D echocardiography continues to remain an appealing imaging modality in heart valve modelling due to its widespread availability and cost effectiveness. Additionally, it avoids the use of contrast agents and ionising radiation, the latter of particular significance for younger patient groups [21–23]. The new generation of 3D TOE platforms boast significantly enhanced temporal and spatial resolutions to derive high-quality volumetric datasets suitable for 3D printing. As such, 3D TOE remains the imaging modality of choice for intraprocedural guidance in mitral interventions and a key source of data for heart valve modelling. CMR is particularly useful for distinguishing soft tissue boundaries and does not require iodinated contrast. Its use is limited by relatively low spatial resolution (making it unsuitable for characterising morphological features of heart valves), incompatibility with metallic implants and time taken for image acquisition [24, 25].

The choice of imaging modality requires careful consideration when planning 3D printing as the accuracy and quality of printed replicas are a direct function of the virtues of the imaging modality employed. 

More recent work has demonstrated the potential of fusion imaging whereby imaging data from multiple modalities can be integrated and then 3D printed to model complex anatomical detail. These techniques seek to offset the limitations of individual imaging modalities and enhance the accuracy and quality of printed replicas [26].

#### Segmentation and virtual modelling

Volumetric imaging data acquired in DICOM format requires processing such that the region of interest can be isolated in a process known as segmentation. There are a constellation of segmentation platforms available including open-source packages such as 3DSlicer, Medical Imaging Interaction Toolkit (MITK) (https://www.mitk.org/wiki/The_Medical_Imaging_Interaction_Toolkit) and ITK-snap (http://www.itksnap.org/pmwiki/pmwiki.php) as well as proprietary platforms such as Mimics (Materialise NV, Leuven, Belgium), Intuition (Tera Recon, Frankfurt, Germany) and Amira (ThermoFisher Scientific, Massachusetts) [27].

Segmentation is often a laborious step with a steep learning curve and requires a high degree of imaging and software expertise. Manual segmentation using editing, cropping and extraction is often arduous, time consuming and has high interobserver variability particularly in the context of mitral valve disease which often features complex anatomy. Several automated segmentation tools have been developed for varying applications such as deriving quantitative valve measures and extracting annular and leaflet geometry for physical printing, or virtual modelling. Automated segmentation tools which often rely on region growing and simple intensity thresholding segmentation methods may struggle in characterising boundaries of cardiac structures of similar intensity profiles such as mitral valve leaflets. Semi-automated segmentation techniques therefore are the most frequently utilised where acquired volumetric images initially undergo automated segmentation followed by manual optimisation by the user [27, 28].

Fully automated mitral valve segmentation however has seen advancements using atlas-based approaches and deep learning-based methods. Atlas-based approaches utilize a large database of manually labelled images, which are used to generate a deformable template which can then be guided to the leaflet geometry. Atlas-based methods relying on sparse geometry or landmarks are potentially limited in the amount of patient-specific detail that can be extracted and may be biased towards the atlas geometry [29, 30]. Deep learning-based approaches have been extensively demonstrated to be highly effective for automated segmentation across many applications. While these methods have been shown to achieve high accuracy for automated mitral valve segmentation, further work is still necessary to validate the generalizability of the approach on larger multi-centre datasets [31]. With continued development, fully automated segmentation offers the possibility of enhancing automation and reducing the time required for segmenting complex cardiac anatomies on the path to future point of care 3D mitral modelling prior to intervention [14, 32].

Optimisation of the post segmentation virtual model is often required using dedicated platforms which enables editing and smoothing. Computer Aided Design (CAD) tools may also be employed to process virtual geometries for example colour coding regions of interest. The virtual rendering is then converted into the file formats (most commonly Standard Tessellation Language, STL format) which are recognised by 3D printers to enable printing a physical replica of the segmented structure. Alternatively, these file formats can be integrated into virtual platforms for computer-aided mitral modeling purposes. [28, 33].

### 3D printing technologies and replica manufacture

#### Printer technologies

**Fig. 2:**
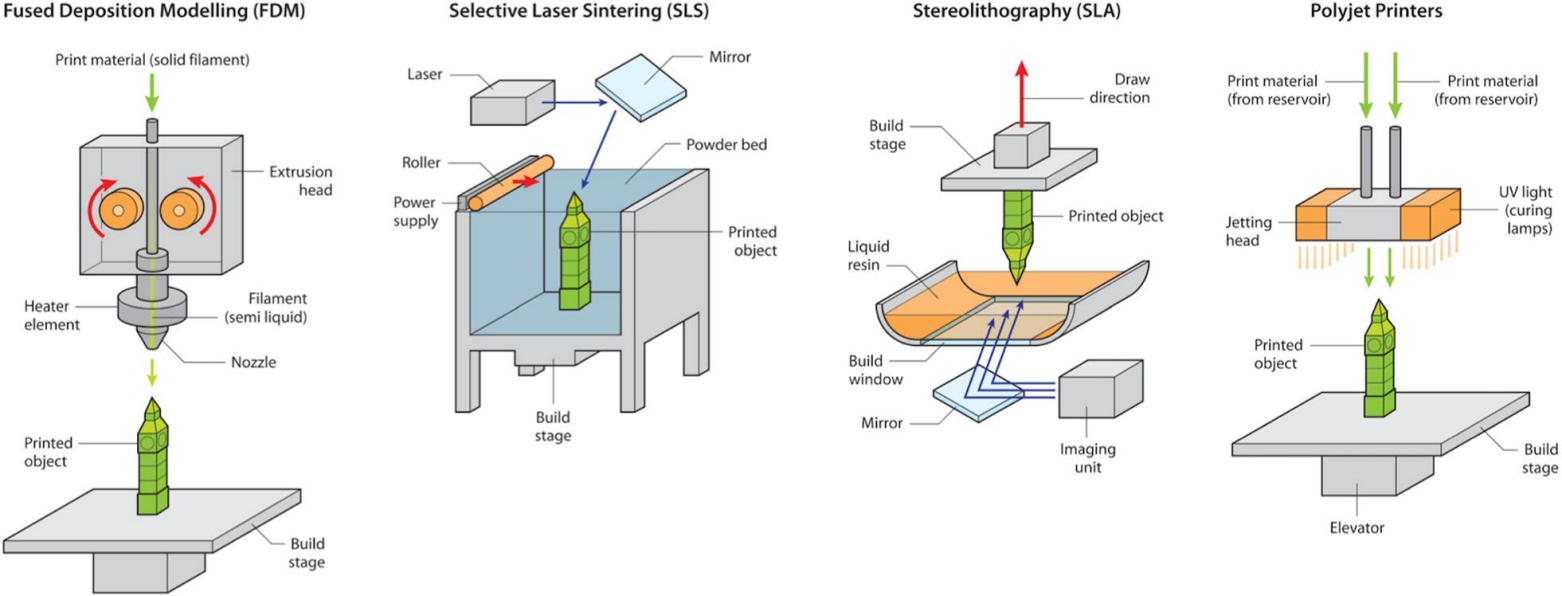
An overview of 3D printer technologies. A schematic representation of common 3D printer technologies currently available. FDM extrudes thermoplastic filaments layer by layer to generate a 3D replica. SLS uses laser to fuse powder-based polymers. SLA uses a liquid photosensitive resin which coalesces on exposure to laser or UV light irradiation. Polyjet printers extrude a photopolymer which solidifies on exposure to a UV light or laser source and offer the ability to combine multiple polymers to generate physical replicas with complex material properties

3D printing technologies are well established having been first introduced by Charles Hull in 1984 by the application of optical technology to rapid prototyping [28]. Figure 2 and Table 1 provide an overview of commonly used 3D printer technologies. Table 1 also offers an outline of the technical aspects and limitations of each 3D printing modality.

**Table 1:** A technical overview of various type of available 3D printer technology

	FDM	SLA	SLS	PolyJet
Resolution	+	+ +	+ +	+ + +
Overall cost	+	+ +	+ +	+ + +
Materials	Polymers [TPU, PLA, PVA]	Photosensitive resins	Wax, Metal, Ceramic Powder	Photo-curable Gel
Finish quality	+	+ +	+ +	+ + +

+  = Low, +  +  = Medium, +  +  +  = High

*FDM* Fusion Deposition Modelling, *SLA* Stereolithography, *SLS* Selective Laser Sintering, *PLA* Polylactic Acid, *TPU* Thermoplastic Urethane, *PVA* Polyvinyl Alcohol

Fusion Deposition modelling (FDM) is the most frequently used technology for cardiovascular 3D printing due to its low cost and ease of use. FDM extrudes molten thermoplastic filaments layer by layer to generate a 3D print. FDM requires the use of a supporting material to provide stability for complex structures during the printing process which can later be removed. FDM printing is limited by low accuracy and offers a poor finish for softer surfaces.

Stereolithography (SLA) uses a liquid photosensitive resin which rapidly coalesces when exposed to laser irradiation or light. SLA printers can generate complex structures with high feature resolution (~ 1.2 mm) however this printing modality is expensive and is restricted by the limited number of available photosensitive resins [33, 34].

Polyjet (PJ) printers are based on the same principle of photopolymer technology used in SLA printers. PJ printers utilize an extrusion process to push out a photopolymer, which is the primary material used for creating the physical model. This photopolymer subsequently solidifies when exposed to laser or light. Furthermore, PJ printers incorporate a photo-curable gel (PolyJet^TM^ by Stratasys) or wax (3D systems) as removable support during the printing process. PJ printers offer high resolution with good quality surface finishes and importantly offer the ability to combine different polymers within a single print. PJ printers therefore show considerable promise in the high-resolution printing of complex cardiac structures with multiple tissue properties such as calcification. PJ printers are currently limited by high cost of hardware and printing consumables.

Selective Laser Sintering (SLS) utilizes a laser to selectively heat and fuse powder-based materials. The SLS process involves converting these materials into particles and subsequently fusing them together in successive layers to form the final 3D printed object. While the primary focus is on powder-based polymers, SLS printers can accommodate a diverse range of materials, including ceramics and metals. Although expensive and resulting in a rough finish, SLS printing allows the generation of highly robust 3D structures [34].

#### Materials technology and requirements for 3D mitral printing

A comprehensive understanding of materials technologies and the properties of native tissues is crucial in creating biomechanically realistic 3D printed models.

The mitral valve leaflets comprise of three layers each containing a heterogenous matrix of constituents with different biomechanical properties to serve specific functional requirements. The atrialis layer is covered with endothelium below which lies connective tissue composed of elastin sheets and laminar collagen. The composition of the atrialis layer allows it to sustain the significant tensile deformation forces associated with systole whilst enabling a degree of recoil in diastole. The presence of smooth muscle in the atrialis layer with its attendant innervation enables some modulation of leaflet stiffness. The glycosaminoglycan rich spongiosa underlies the atrialis layer and contains irregularly arranged collagen. The spongiosa layer helps resist the compressive forces from mitral leaflet coaptation by retaining water. The Fibrosa layer contains densely packed collagen fibres aligned parallel to the leaflet free edge and flexibly crimped. At the site of chordal insertion, the fibrosa transitions from a planar to a cylindrical arrangement which prevents leaflet kinking by enabling a gradual transition of forces from the chordae and leaflets. The chordae tendineae are complex structures featuring an elastin sheath housing a cylindrical collagenous chord [35–38].

Table 2 summarises the mechanical properties of common print materials and native cardiac tissue. Conventional polymers such as polylactic acid (PLA) and thermoplastic urethane (TPU) have proven useful in manufacturing affordable and straightforward static models for procedural appreciation of anatomy and patient education. However, these polymers fall short in replicating the complex dynamic architecture of mitral tissues. This is further complicated by the heterogeneity of pathologies resulting from age, co-morbidities, and variable calcium deposition. Furthermore, modelling mitral interventions also requires accurate replication of device-native tissue interactions or device-device-tissue interaction in the instance of valve-in-valve procedures. Although polymer-based models with uniform tensile characteristics may simulate soft tissue behaviour under low strain, significant behavioural variance is observed under larger deformation. The biomechanical properties of healthy and pathological human tissue also remain to be fully elucidated therefore limiting the development of realistic 3D printing materials. Direct 3D printing of realistic mitral valve replicas therefore remains challenging particularly as the ability to integrate multiple materials within a replica has hitherto been limited [39–41].

**Table 2:** An overview of mechanical properties of common print materials and native cardiac tissue

Material**	Print method	Elastic modulus (MPa)	Shore hardness	Cost
TPU	FDM	26	95 (Scale A)	+
PLA	FDM	3000–4000	83 (Shore D)	+
ABS	FDM	2200	76 (Shore D)	+
VeroWhite	PolyJet	2000–3000	83–86 (Shore D)	+ +
TangoPlus FLX973	PolyJet	1.8–2.4	6–62 (Shore A)	+ + +
Agilus Black	PolyJet	2.4–3.1	30–35 (Shore A)	+ + +
EcoFlex 00–30	Injection moulding on 3D printed model	1.4	00–30	+
MoldStar 15	Injection moulding on 3D printed model	2.7	15 (Shore A)	+
Native valves	–	1.0–1.3	–	–
Heart muscle	–	0.08	–	–

The elastic modulus is defined as an object’s resistance to indentation of a given material with two commonly used scales; Type A and Type D. *PLA* Polylactic Acid, *TPU* Thermoplastic Urethane; *ABS* Acrylonitrile Butadiene Styrene, *FDM* Fusion Deposition Modelling, *MPa* Mega Pascals

The development of flexible elastomeric materials such as HeartFlex and TangoPlus along with the advent of polyjet printers which enable manufacture of complex models by blending printing materials (termed metamaterials) may herald a paradigm shift in towards the direct manufacture of biomechanically realistic mitral replicas capable of being integrated into functional and static mitral modelling for prospective interventions. Wang et al. were one of the first to demonstrate the feasibility of using metamaterials to realistically simulate the stress–strain properties of human valvular tissue. The authors used TangoPlus (simulating human extracellular matrix) embedded with VeroBlackPlus (simulating stiff collagen fibres within extracellular matrix) either in sinusoidal or double helix geometries. They then compared the stress–strain relationship of human aortic tissue to TangoPlus alone and the metamaterial incorporating a sinusoidal wave geometry to find the metamaterial to emulate human aortic tissue more closely. The authors also demonstrated that stress–strain relationships of the metamaterials can be ‘tuned’ by changing the characteristics of the ingrained double helix or sinusoidal patterns [41]

In the mitral domain, Vukicevic et al. demonstrated the feasibility of patient-specific manufacture of the entire mitral valve apparatus using metamaterials and their ability to permit implantation of transcatheter devices. Significantly, they attempted to simulate the material properties of surrounding structures and the unique layered microanatomy of mitral leaflets by incorporating different shores (hardness) of TangoPlus. Calcification was modelled using very hard VeroWhite material. The authors compared stress–strain relationships of healthy porcine mitral leaflets to that of two individual grades of TangoPlus and a combination of the two grades to find that TangoPlus was able to model the stress–strain relationship of healthy porcine leaflet tissue within the zone of physiological function and at the transitional region where collagen fibres are recruited [40]. More recently, Vukicevic et al. demonstrated that combinations of the photopolymers Agilus and Rigur (Stratasys, Minnesota, USA) were able to approximate the mechanical properties of various components of the mitral valve apparatus (leaflets, chordae and annulus) at physiological levels of strain thus suggesting that multi-material 3D printed patient-specific replicas may be capable of modelling the deformation forces that act upon devices used for mitral valve interventions. As such 3D printed replicas fabricated from metamaterials have the potential to be utilised in studying tissue-prosthesis interactions with possible implications on future prosthesis choice and sizing [42, 43].

Although polyjet printing and the development of metamaterials offer promise in realistically simulating mitral behaviour, such technologies are currently expensive and require specialist expertise greatly limiting their wider clinical translation. Moreover, such rigid mitral replicas are unable to reproducibly simulate complete mitral valve tissue properties and therefore essentially serve as morphological displays precluding functional replica evaluation. As such, ‘soft’ deformable silicone based mitral replicas derived from moulding techniques have been favoured for dynamic mitral modelling and simulation. Moulds are often 3D printed and have patient-specific properties however they lack the anatomical precision of directly 3D printed mitral replicas and therefore offer a heuristic approach to modelling [40, 41, 43]. Daemen et al., were one of the first to demonstrate the feasibility of reproducibly creating patient-specific deformable silicone replicas which allow surgical instrumentation for the purpose of mitral modelling and simulation [44]. The material properties of silicone-based replicas have been shown to approach that of mitral tissue (see Table 1). Indeed, Yang et al. compared the Shore hardness and Elastic modulus of TangoPlus and 3 other silicones (Silicone 5A, Silicone 10A and Silicone 20A; Hong Cheng Silicone Products factory, Guangdong, China) to find Silicone 10A to have material properties akin to valve tissue [45]. Whilst mould derived silicone models may be able to emulate mitral tissue properties to a certain extent, they lack the precision that direct 3D printing offers. Future advancements in printer and materials technologies hold the potential for manufacturing mitral microarchitecture using multiple materials. ‘Soft’ mitral replicas also require housing within support structures to enable incorporation into simulators. This may require modification of the original imaging data following segmentation using CAD software therefore distorting patient-specific anatomical features in the resulting replica.

## Application of 3D printing in modelling mitral valve interventions

The translation of 3D printing technology in modelling mitral interventions can broadly be divided into the use of static mitral replicas and in vitro functional platforms.

Static replicas seek only to replicate patient-specific anatomy and can be used as adjunctive aids in the comprehension of complex mitral anatomy, procedure planning and patient education. In vitro functional platforms seek to replicate patient-specific mitral anatomy and the haemodynamic milieu of mitral disease. Functional platforms can serve as research testbeds to study mitral pathophysiology and evaluate novel mitral technologies. They also have the potential to be used for dynamic procedure planning and immersive patient-specific simulation training [46].

### Procedural planning

#### Static 3D-printed mitral replicas

**Fig. 3:**
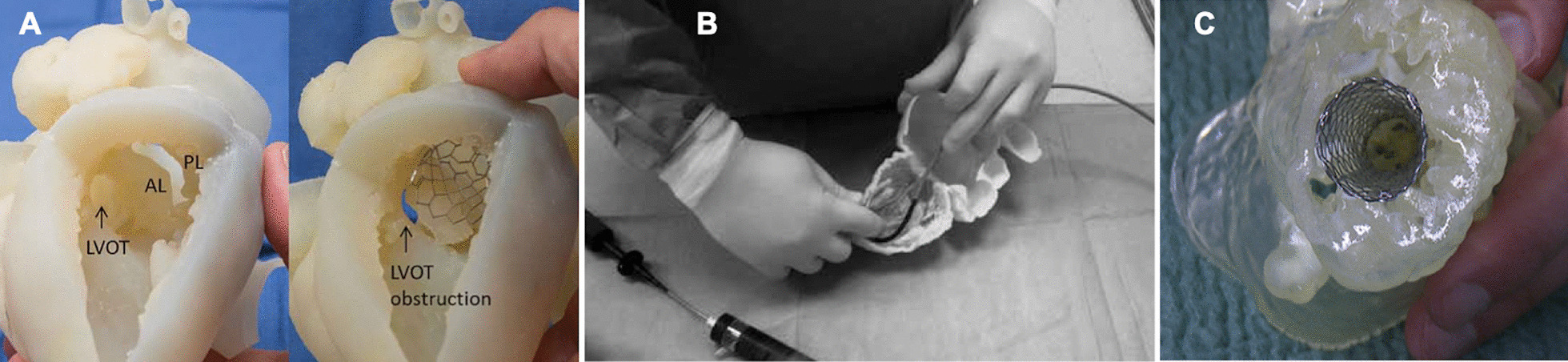
Application of static patient-specific 3D printing in procedural planning. **A** 3D printed heart model to assess the risk of LVOTO in patients undergoing TAVI valve in mitral annular calcification procedure. *AL* anterior mitral leaflet, *PL* Posterior Mitral leaflet. Adapted from Sabbagh et al. [55]. B Demonstrates the use of a static 3D printed mitral replica for sizing the guide catheter curve within the left ventricular cavity in a patient undergoing direct percutaneous annuloplasty using the Mitralign system. Adapted from Dankowski et al. [52]. C demonstrates an implanted LOTUS TAVI prosthesis within a 3D printed patient-specific mitral replica which was used to aid procedural planning for transapical transcatheter mitral valve implantation. Adapted from Ren et al. [54]

Static 3D-printed replicas offer haptic and spatial appreciation of complex anatomy, with potential dividends in procedural planning, especially given the steep learning curves associated with mitral interventions [46, 47]. Moreover, such patient-specific 3D printed replicas can potentially guide key aspects of interventional strategy such as device sizing (Fig. 3). Mahmood et al. demonstrated the feasibility of 3D printing rigid patient-specific models of the mitral annulus pre-and post-surgical repair [48]. Although not validated with the size and shape of the native annuli, this early work demonstrated the potential of static models to aid the selection and sizing of mitral annuloplasty rings which relies heavily on surgical judgement and experience. Similarly, Witschey et al. demonstrated the feasibility of 3D printing annular and leaflet morphology in systole and diastole for normal and diseased mitral valves [21]. This work was limited by the lack of quantitive validation with native tissue dimensions on TOE which is a prerequisite for clinical translation. Shirakawa et al. have since demonstrated that 3D printed replicas of patients with mitral valve prolapse had acceptable dimensional differences when compared to actual native tissue evaluated intra-operatively [49]. Whilst much of the work in static mitral modelling shows promise, it is crucial to note that these studies are proof of concept work and therefore cannot yet be clinically translated. Moreover, given the dynamic and complex geometry of the mitral valve apparatus, the accuracy of measurements and prostheses sizing derived from static 3D printed mitral replicas is highly questionable.

Although of value, rigid 3D printed mitral replicas are bound by the current limitations of materials technology. As such, directly 3D printed replicas currently preclude surgical instrumentation such as the deployment of annuloplasty rings, suturing and leaflet resection limiting their translational value for surgical planning and simulation. ‘Soft’ silicone based mitral replicas derived from moulding techniques can simulate the gross properties of mitral tissue whilst being sufficiently deformable to facilitate surgical or trans-catheter instrumentation. The properties of ‘soft’ mitral replicas may therefore lend themselves more favourably to procedural planning. Indeed, in proof of concept work Nia et al. were able to simulate the prospective surgical repair of a P2 prolapse within a static bench top simulator using a deformable patient-specific mitral replica derived from 3D TOE. The authors were subsequently able to undertake successful real-life surgical repair with no alterations to the simulated surgical technique. Interestingly, note was made of anterior leaflet prolapse on intraoperative saline testing post-repair. This was not apparent on the pre-operative TOE or the simulated model and deemed to be pseudo prolapse which was confirmed on intraoperative TOE. In some instances, junior surgeons have been compelled to intervene upon leaflet pseudo prolapse adversely impacting outcomes. The authors argued that this event revealed the potential value of integrating physical 3D modelling with pre-operative imaging in intra-procedural decision making [50]. Similarly, Yang et al. showed correlation between morphological and mitral dimensions obtained on patient-specific silicone replicas and those obtained intraoperatively. Interestingly, the authors also found morphological parameters such as coaptation depth and leaflet/annulus ratio in repaired replicas to be associated with residual post operative MR therefore suggesting that these replicas can not only be used for procedural planning but also modelling post-repair outcomes [51].

Unlike surgical interventions, transcatheter procedures preclude intraprocedural examination of mitral pathologies therefore depriving interventionalists the opportunity to alter procedural strategy at the point of intervention, making such interventions less predictable than their surgical equivalents. As such, there is arguably greater reliance on preprocedural planning and greater exposure to the limitations of cardiac imaging in transcatheter interventions compared to surgical techniques. Moreover, given their high-risk nature, patients undergoing transcatheter interventions are less likely to be offered surgical ‘bail-out’ in the event of complications. There has therefore been an evolving interest in integrating 3D printing and modelling into transcatheter Heart Team procedure planning for increasingly complex transcatheter mitral valve interventions. Dankowski et al. were one of the first to apply 3D printing techniques as an adjunct to procedure planning for percutaneous mitral annuloplasty using a static phantom derived from MDCT. They found the printed replica enabled them to accurately examine the underside of the mitral valve and identify the precise location, number and size of papillary muscles which has a bearing on initial crossing wire placement. The degree of basal dilatation was also apparent on the replica therefore guiding optimal placement of Mitralign pledgets. The derived model also allowed characterisation of parameters such as the aortic entry angle into the LV and analysis of subvalvular apparatus to guide equipment selection and placement [52]. More recently static 3D printed mitral phantoms have been used as an adjunct to guide complex and high risk transcatheter mitral interventions. Indeed, Little et al. used a CT derived 3D printed multi-material model of mitral leaflets and sub-valvular calcium deposition to guide the sizing of an occluder device to seal a posterior mitral leaflet perforation [53]. Ren et al. used a MDCT derived 3D printed model of mitral stenosis to confirm prosthesis sizing and assess the feasibility of transapical transcatheter mitral valve replacement using a LOTUS tissue heart valve [54].

There are several key limitations precluding the widespread clinical translation of static 3D printed mitral replicas in procedural planning. The materials employed for printing do not entirely mimic native tissue and therefore the impact of dynamic device-host interaction cannot be accurately modelled especially due to the static nature of modelling which has implications for their use in crucial areas such as sizing prostheses. There is no standardised or validated technique available to manufacture mitral replicas which greatly limits the translational potential of research within this area. As such incorporation into procedure planning especially in complex interventions requires careful consideration. Clinical translation is also precluded by cost, time taken for image processing and printing as well as the lack of widespread expertise in this arena.

The integration of static 3D printed mitral phantoms with complementary transformative technologies however, have the scope to greatly enhance their combined efficacy and translational potential.

#### Computer assisted mitral modelling

TMVR technologies are in their nascency with a myriad of novel devices available for implantation. Moreover, transcatheter valve-in-MAC and valve-in-ring procedures utilise tissue heart valves tailored for transcatheter aortic valve implantation (TAVI).

3D computer assisted modelling (3D-CAM) platforms enable the derivation of static virtual anatomical 3D models of patient-specific mitral pathology using volumetric imaging data. Derived virtual models can then be manipulated to evaluate complex anatomy and assist in aspects of procedural planning such as sizing of prosthesis, predicting the risk of significant complications, and devising appropriate interventional management strategies. Patient-specific static 3D printing has been used to complement 3D-CAM [55–58].

**Fig. 4:**
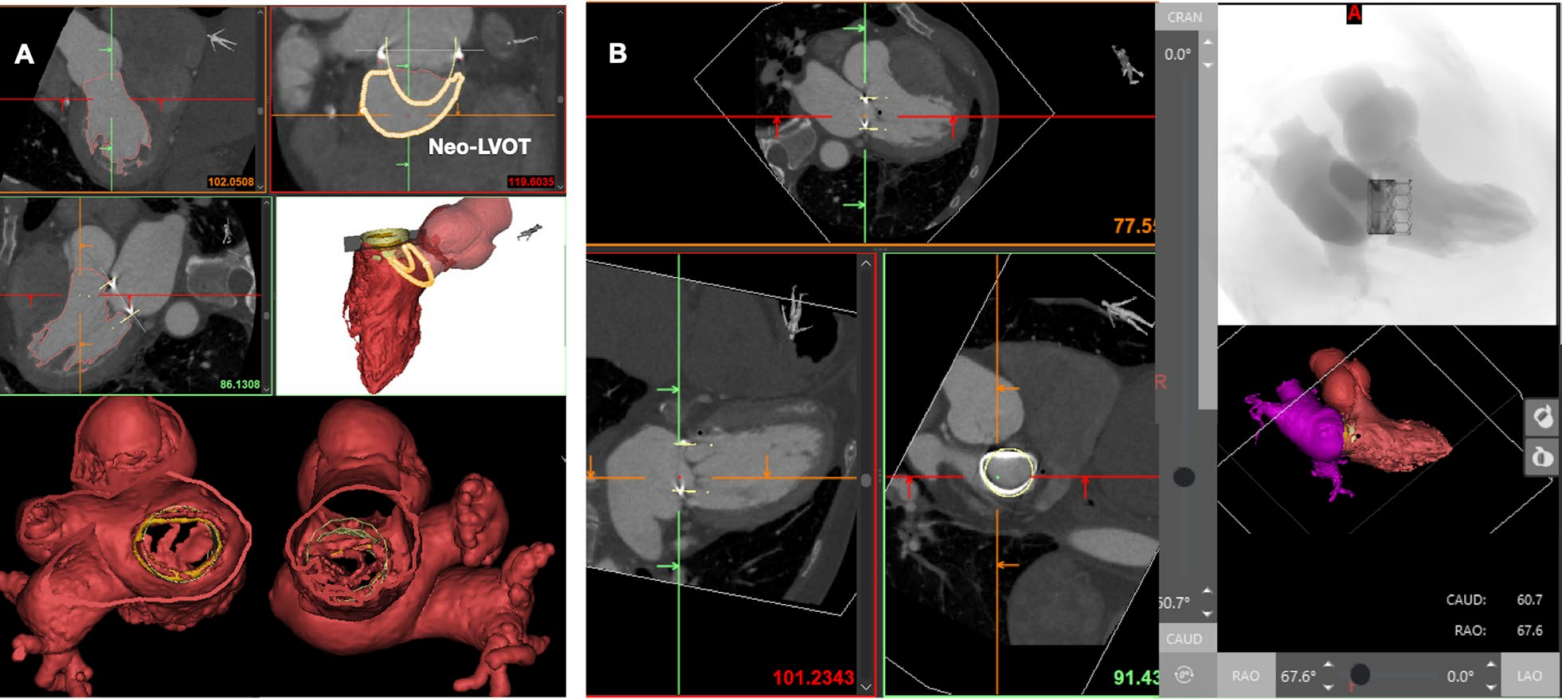
3D computer assisted modelling (3DCAM) for transcatheter mitral valve intervention. Patient-specific Multidetector CT data is segmented and the STL file is used to generate a virtual 3D model of patient anatomy which can be evaluated by the Heart Team in multiple planes and used to simulate the trans-catheter mitral valve replacement to assess the neo LVOT and risk of LVOT obstruction (**A**). The imaging data can also be used to identify optimal fluoroscopic procedural views (**B**)

TMVR displaces the anterior mitral valve leaflet and changes the geometry of the LVOT with the resulting morphology termed the neo-LVOT. TMVR carries a risk of LVOT obstruction (LVOTO) which can be fatal. 3D-CAM is increasingly being adopted for procedural planning and predicting the risk of LVOTO and has shown good correlation between modelled and real-world procedural outcomes [59] (Fig. 4). 3D printed mitral replicas used in conjunction with 3DCAM have been employed successfully in modelling the risk of LVOTO. Specifically, they offer interventionalists a complete spatial appreciation of anatomy with the proposed device in situ and the ability to physically evaluate the virtually modelled LVOTO risk. This can facilitate the derivation of interventional strategies such alcohol septal ablation or laceration of the anterior mitral valve leaflet (LAMPOON) procedures to minimise the procedural risk in the event of LVOTO. Indeed, Sabbagh et al. demonstrated the feasibility of using CT based 3DCAM and 3D printing for simulating valve sizing, device apposition, risk of paravalvular leak (PVL) and LVOTO in 8 patients undergoing TMVR for MAC [55]. 3DCAM was found to simulate LVOTO in one patient who then underwent preventative alcohol septal ablation with a subsequently successful TMVR (TAVR-in-MAC). In another case the authors were able to not only physically model the impact of valve deployment on a multi-material 3D printed heart model which resulted in LVOTO, but also model the impact of proposed pre-deployment anterior mitral valve resection and the degree of resultant PVL. In a recent larger multi-centre study, Wang et al. demonstrated 3DCAM used in conjunction with 3D printing to be an accurate and reproducible method to predict Neo-LVOT dimensions and risk of LVOTO in 38 patients undergoing balloon expandable TMVR [59]. Although limited by small sample sizes, non-standardised protocols and the lack of tissue mimicking 3D printing, such work demonstrates the potential of integrating disruptive technologies in procedure planning for transcatheter mitral interventions and the versatility of 3D printing.

In addition to procedural risk prediction and mitigation, 3DCAM can also be employed to derive an enhanced understanding of patient-specific anatomy and optimise interventional strategy for those undergoing transcatheter mitral interventions. For example, 3DCAM can be used to derive an enhanced appreciation of the anatomical relations of the inferior vena cava, interatrial septum, and mitral annulus which can then be 3D printed to provide the operator a physical evaluation of the interatrial puncture site and an impression of the impact of patient-specific anatomy on delivery catheters and other equipment [57, 59]. 3DCAM platforms can also assist in the pre-procedural identification of optimal fluoroscopic projections, which could enhance operators’ understanding of patient anatomy and reduce contrast and radiation dosage [60–62].

Despite the considerable advantages associated with incorporating 3DCAM in transcatheter mitral intervention planning, it remains a static modelling modality unable to simulate the complex dynamic anatomical changes and flow dynamics inherent to mitral valve diseases. As such, the derived modelling information must be considered within the wider scope of Heart team procedure planning and integrated with other imaging modalities and the clinical context.

#### 3D-printing based dynamic mitral modelling

Mitral valve pathologies are unique in that they can only be fully appreciated within a haemodynamic environment, therefore immediately limiting the value of static physical mitral modelling. As such dynamic ex-vivo models comprising of animal mitral valve tissue integrated into mock circulatory systems (MCS) seeking to emulate human cardiac haemodynamics form the mainstay of functional mitral modelling and allow evaluation of research hypotheses, novel transcatheter devices and operator simulation training [63].

**Fig. 5:**
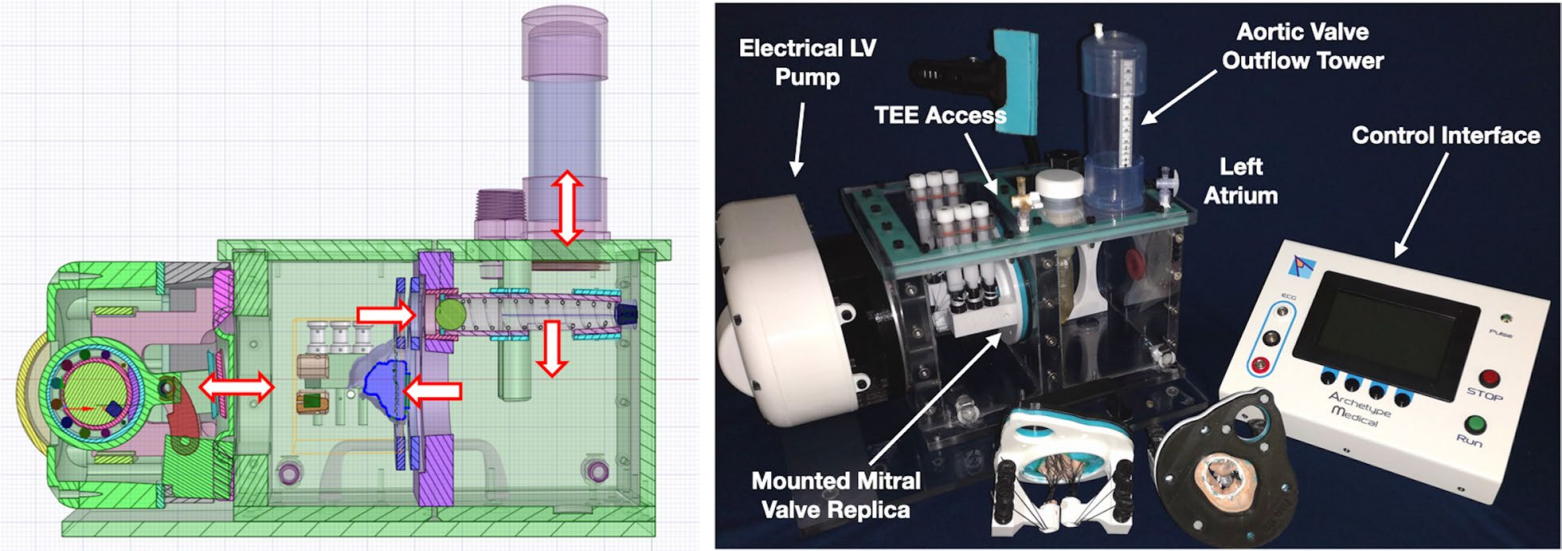
An example of a Mock Circulatory System (MCS). Left ventricular haemodynamics are simulated by electrical motor which can be tuned by a control interface to modulate physiological variables such as heart rate and stroke volume. Blood mimicking fluid (tap water in this system) is driven through the modelled left heart chambers. A spring within an aortic outflow tower (AV outflow) can be altered to set afterload. A patient-specific silicone valve replica is placed within the system and evaluated using transoesophageal echo. Mitral pathology is simulated by tuning the tension on the chordae of the patient-specific valve replicas [65]

The constituents of an MCS are outlined in Fig. 5. Briefly, such systems usually comprise of a drive unit to simulate the ventricular function, a blood mimicking fluid, and a system for morphological and/or haemodynamic characterisation which may be through catheter-based monitoring, Doppler ultrasound, particle image velocity measurement or 4D flow MRI [40]. Dynamic ex-vivo mitral models are not patient-specific and are limited by cost and ethical considerations in the use of animal tissue making them unsuitable for high volume research.

The advent of 3D printing offers the prospect of incorporating and dynamically modelling patient-specific mitral valve replicas within an MCS. Such a system however is reliant upon the manufacture of mitral replicas exhibiting the biomechanical properties of human valves and accurate simulation of the haemodynamic milieu of mitral pathologies. The current status of materials and printing technologies preclude direct printing of biomechanically realistic mitral replicas. Patient-specific mitral valves created by casting silicone replicas over rigid 3D printed models have been shown to provide a heuristic approach for modelling mitral pathologies within a mock circulatory system environment. This approach arguably can be seen as a bridging technology until materials technologies are sufficiently mature to allow direct realistic mitral printing.

Mashari et al. demonstrated the feasibility of the haemodynamic evaluation of a 3D printed mitral valve after TMVr within an MCS. The authors were able to derive pressure half time values from continuous wave Doppler and chamber pressures through pressure monitoring catheters. The study was limited by the ability of the MCS to only generate sufficient pressures to allow diastolic modelling [64].

**Fig. 6:**
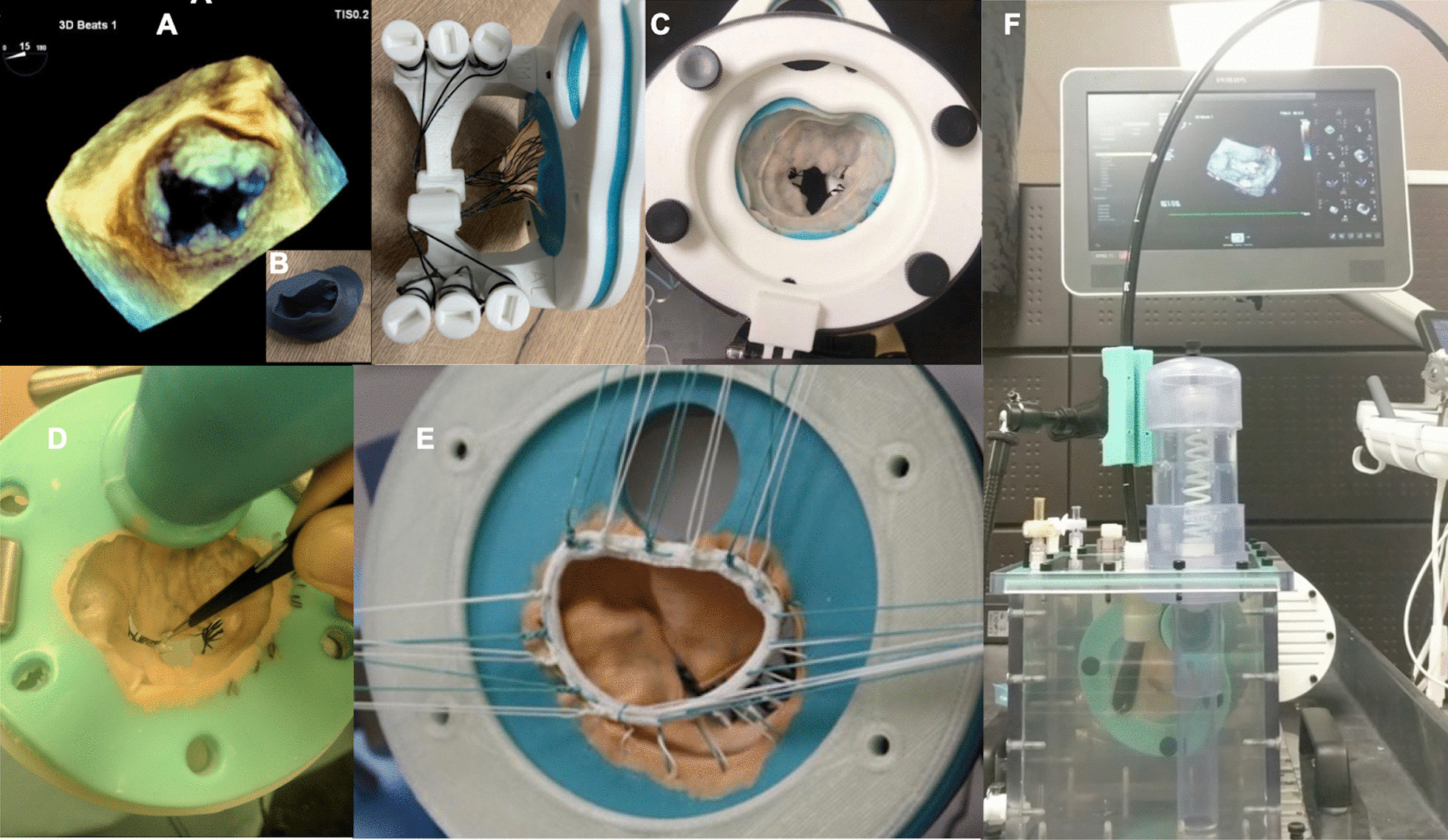
Silicone based patient-specific mitral valve replicas using an indirect injection moulding technique. Image **A** demonstrates a 3D echo dataset used to create a patient-specific rigid mould of the mitral valve in diastole **B** into which tissue mimicking silicone is applied. Image **C** shows incorporation of the silicone made mitral valve replica into an assembly that allows integration of the model into the mock circulatory circuit. Valve replicas can be modified per the proposed interventional strategy such as edge-edge mitral leaflet repair **D** or placement of an annuloplasty ring (**E**). These replicas can then be mounted and evaluated using transoesaphageal echo within a mock circulatory circuit (**F**)

In more extensive contemporary work, Ginty et al. semi-automatically segmented 3D TOE data in diastole to derive patient-specific 3D printed PLA moulds of the atrial mitral valve surface. Silicone (Ecoflex 00–30, Smooth-On Inc, East Texas, PA) was then used to create a patient-specific mitral valve assembly with incorporated gauze fabric shaped to anterior and posterior mitral leaflets as well as flayed braided Dacron threads to replicate six chordae tendinae. Gauze fabric provided additional tensile strength to adequately mimic mitral leaflets, prevent dehiscence within simulated haemodynamic conditions and allowed the application of sutures or clips to simulate mitral valve interventions (Fig. 6). Braided Dacron adequately mimicked the flexibility of chordae whilst bearing sufficient strength to operate under realistic haemodynamic conditions. Patient-specific mitral valve assemblies were then incorporated into a purpose-built benchtop MCS (Fig. 5) simulating the left heart and evaluated in vitro using 3D TOE. The MCS design is such that it enables the user to interactively adjust the braided dacron’s length to mimic patient’s mitral regurgitation pathology. The authors demonstrated no difference in quantitive and qualitative anatomical and MR parameters on in-vitro modelling when compared to those obtained in-vivo therefore demonstrating the feasibility of the platform to heuristically model mitral pathologies [65]. This work serves as apt example of the potential of indirect injection-moulding techniques to manufacture functional and deformable patient-specific mitral replicas. These are capable of being integrated within a MCS for dynamic mitral modelling within a simulated physiological environment. In a separate study Ginty et al. were also able to demonstrate the feasibility of this platform to model mitral post repair states [66].

The availability of a high-fidelity patient-specific mitral valve simulator has significant transformative potential with the scope for integration into Heart Team workflows surrounding patient selection and procedure planning and thus potentially enhancing procedural outcomes and patient safety. Moreover, such a platform has the potential to be integrated with other transformative technologies further expanding its utility. 

Although promising, dynamic patient specific mitral modelling remains nascent and a considerable distance away from clinical translation. Indeed, studies in this arena are limited and only demonstrate feasibility, therefore larger studies are required for further validation prior to which caution must be applied to its application in clinical practice. Other practical limitations include the time taken to fabricate silicone based mitral replicas and that replica leaflet thickness is greater than native tissue. Moreover, any progress in this arena is contingent on advances in 3D printing technologies and the ability of MCS platforms to accurately model human haemodynamics.

### Operator training and education

Surgical and transcatheter mitral interventions are technically complex and associated with significant intraprocedural neurocognitive demand thereby resulting in steep learning curves which are closely related to procedural volumes. Indeed, cardiac surgeons have been shown to require between 75–125 procedures to overcome the learning curve for minimal access mitral valve repair [67, 68]. Importantly, studies have demonstrated a close relationship between procedural volume and outcomes with evidence indicating surgeons need to undertake at least 25 mitral valve repairs annually to sustain acceptable outcomes once the learning curve has been surmounted [68]. Similar findings are noted in the transcatheter realm with inflection points on institutional learning curves for procedural time, success, and complications apparent after 50 trans-catheter edge-to-edge repair (TEER) procedures and continued improvement noted up to 200 cases [69]. Interestingly after adjusting for multivariate confounders, only procedural time was found to be significantly associated with institutional volume suggesting a crucial role for patient-selection in determining outcomes which is itself dependent on institutional experience.

Currently surgical and transcatheter training is dependent on the apprentice model of supervised learning and the principle of ‘see one, do one, teach one’ [70, 71]. The efficacy of this model of training however is becoming increasingly eroded by the pressures of modern medicine namely an increasingly frail population with complex co-morbidities, the advent of novel interventions and stretched training budgets. There is often insufficient time to substantively teach technical skills, little accommodation for varying learning styles and limited exposure to complex cases or adverse events [72]**.** Moreover, given their complex nature, mitral interventions are typically undertaken in specialist centres with relatively low volumes further straitjacketing training opportunities. In the case of transcatheter mitral interventions many senior interventional cardiologists are still in the process of transitioning from coronary intervention and TAVI therefore further reducing the scope of technical training available for junior trainees.

Simulation training may offer junior trainees the opportunity become proficient in basic technical skills before graduating onto an apprentice style model whilst more senior operators can acquire the basic skills required for novel procedures. Simulation training is well established in the surgical domain and has been shown reduce the learning curve particularly in laparoscopic surgery [73]. Significantly, there appears to be transferability of skills acquired in simulation environments to the real-world operative setting [72].

Low-fidelity, low-cost physical simulation platforms for surgical mitral valve interventions have been available for some time [74]. Given the increasing complexity of mitral interventionss and the limitations of the current apprenticeship model of interventional training, there is pressing need for validated, high-fidelity mitral simulation training platforms which can be integrated within standardised specialty wide training programmes enabling objective temporal evaluation and feedback on trainee skills.

Important progress has been achieved in this endeavour with direct or indirect 3D printing of patient-specific mitral replicas incorporated into either static (e.g. Mitral Valve Base [LifeLike BioTissue Inc, Ontario, Canada] or the MA-TRAC High fidelity minimally invasive mitral valve repair simulator [Maastricht Trading Company Inc, Netherlands) or a dynamic mock circulatory system. 3D printing has also been utilised to manufacture precision components for animal based ex-vivo simulators which can be adopted for training.

**Fig. 7:**
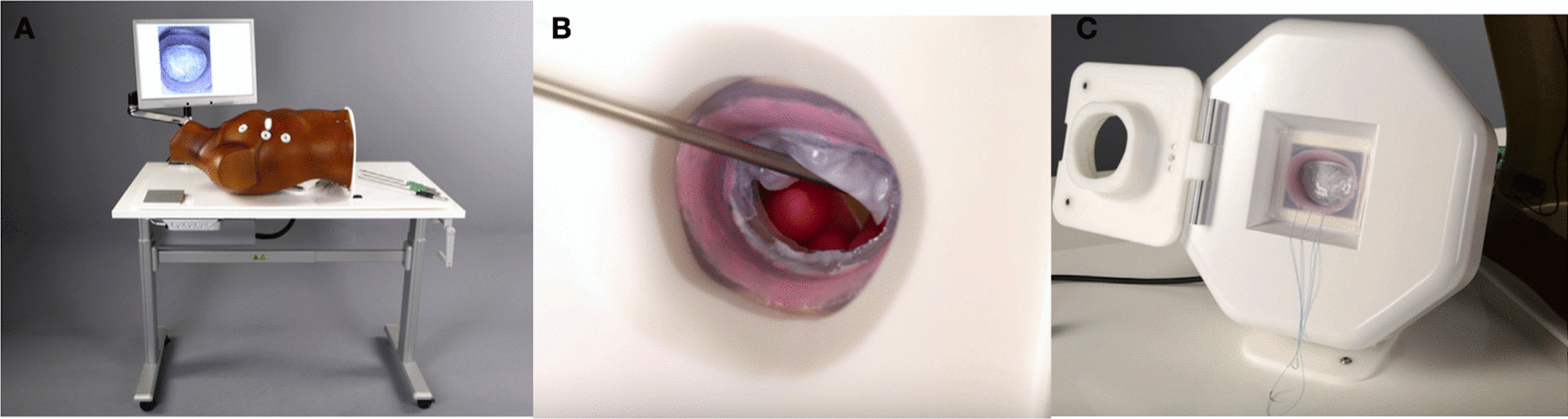
A model thoracic torso with access ports for  simulating minimally invasive mitral repair (**A**) housing a silicone cast mitral valve replica (**B**) mounted within a feedback system assessing suture depth and width (**C**). Adapted from Sardari Nia et al. [75]

Recent work by Nia et al. with the support of the European Association of Cardiothoracic Surgery is an apt example of how 3D printing and silicone based mitral modelling can facilitate efforts towards the systematic development of a validated mitral simulator which can be incorporated into a wider standardised training programme. The investigators conceived, manufactured, and validated a bench top static minimally invasive mitral simulator based on simulation requirements set out by a panel of experienced mitral surgeons. The simulator comprised of a model thoracic torso with robotic and endoscopic access ports. The torso housed a silicone casted generic mitral valve replica mounted within a 3D printed left ventricle and featured a real-time feedback system for suture depth and width (Fig. 7). The authors used a weighted silicone mix of Sorta-Clear 18 (Smooth On Inc, Easton Pa) and Slacker (Smooth On Inc, Macungie Pa) which was found to realistically simulate the suture experience in mitral valve repair whilst maintaining the integrity of the replica as well as allowing objective, predictable measurement of suture depth and width. A workflow to manufacture patient-specific silicone mitral replicas using 3D TOE data was derived to facilitate case specific simulation if required [75]. In a subsequent study, the simulation platform was integrated within a two-day air pilot concept-based training course for endoscopic mitral repair and was found to enhance the accuracy and speed of suturing independent of the grade of operator due to its ability to provide reproducible metric-based performance feedback [76].

**Fig. 8:**
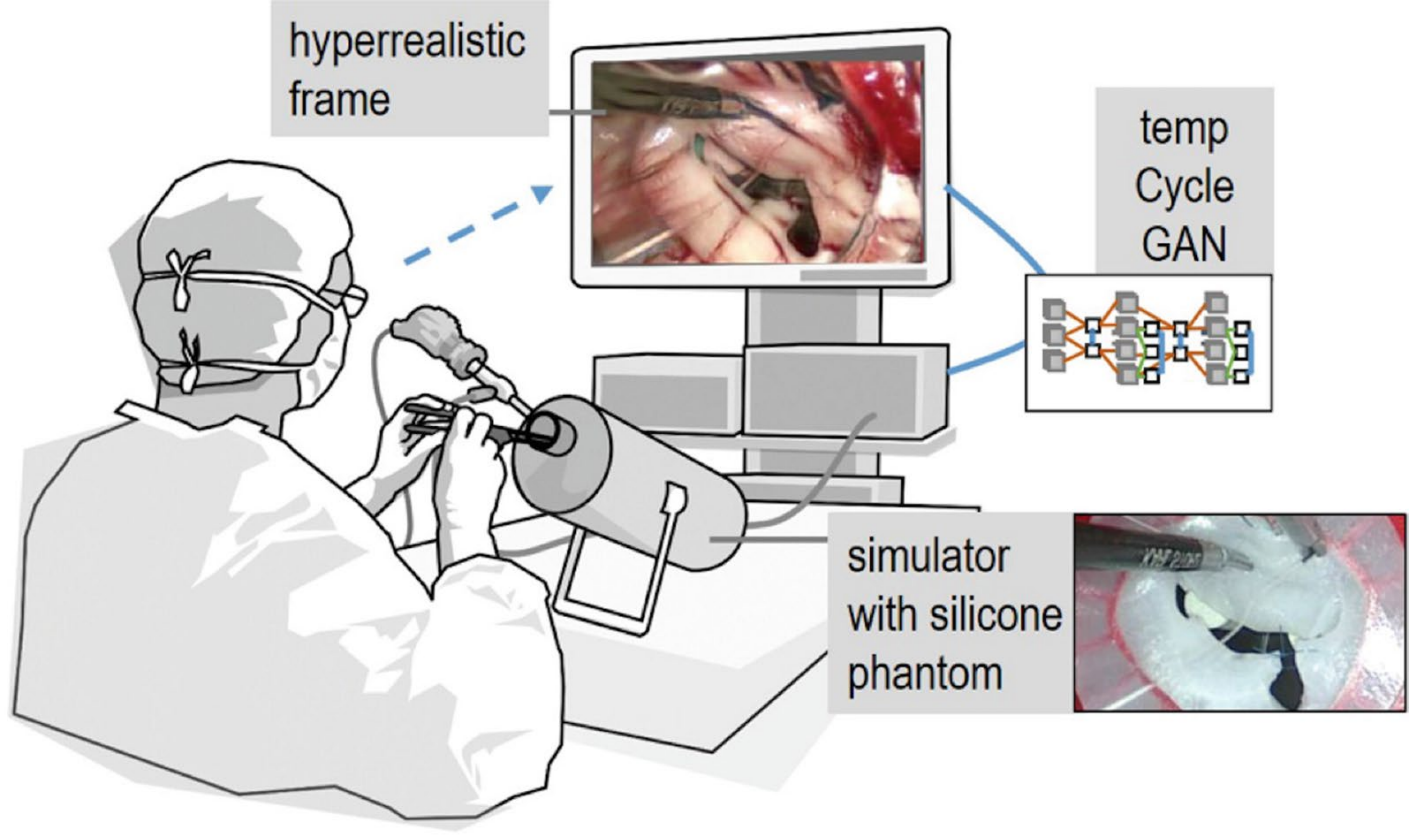
Static 3D printed mitral replicas are sufficiently versatile to be integrated with other disruptive technologies such as machine learning and extended realities. Generative adversarial networks (GAN)—a form of machine learning- were used to map patterns learned from intra-operative video sequences in endoscopic mitral valve repair onto the video stream captured during training on non-dynamic mitral surgical simulator using silicone mitral replicas to provide a hyper-realistic training experience. Adapted from Engelhardt et al. [77]

The integration of 3D printing with other transformative technologies to derive hybrid patient-specific simulation platforms may harbour considerable future translational potential through offsetting limitations of individual technologies, conceivably resulting in a highly optimised platform. Engelhardt et al. demonstrated the feasibility of such a paradigm by first developing a standardised workflow incorporating 3D printing techniques to manufacture patient-specific silicone mitral valve phantoms which included the papillary muscles and chordae tendinae. The authors integrated these replicas within a non-dynamic commercial endoscopic mitral valve repair simulator and demonstrated it to be valuable for training junior surgeons and for patient-specific procedural rehearsal for senior surgeons [77]. In further work, Engelhardt et al. then used generative adversarial networks to map patterns learned from intraoperative video sequences in endoscopic mitral valve repair onto the video stream captured during training with silicone mitral replicas for more immersive simulation of the intraoperative domain [78]. This machine learning method seeks to generate an impression of the modelled mitral pathology such that parts of the physical phantom that appear unnatural are replaced by realistic appearances (Fig. 8). This concept is termed hyperrealism which is a sub-form of augmented reality. Although such platforms integrating transformative technologies show promise, it is important to recognise, that the efficacy of any such platform for patient-specific modelling is a function of the quality of the cardiac imaging from which they have been derived.

The increasing global volume of transcatheter mitral interventions (primarily edge-to-edge repair) has spurred the development of dedicated physical high-fidelity simulators in this arena with a view to training the next generation of interventional cardiologists and facilitating the transition of the current generation from TAVI to transcatheter mitral interventions. Operators undertaking TEER interventions not only require considerable dexterity but also simultaneously need to process and integrate real-time information from 3D TOE and fluoroscopy whilst maintaining close interaction with a multi-disciplinary catheterisation lab team to deploy repair devices safely and successfully within a beating heart. As such the development of effective high-fidelity physical simulators beyond ex-vivo animal models in this arena is fraught with challenges and in its nascency with 3D printing set to assume a pivotal role. Early work by Vukivecic et al. demonstrated the feasibility of successfully deploying and securing a Mitraclip onto a multi-material patient-specific 3D printed mitral replica- an important prerequisite for any substantive simulator [43].

Meanwhile, Laing et al. validated the ability of a benchtop static simulator comprising of a patient-specific silicone-based heart model (manufactured using 3D printed moulds) mounted within a cylindrical container with port access to accurately display anatomical features and equipment on TOE and fluoroscopy to simulate septal puncture—a particularly demanding and crucial element of mitral TEER [79]. More recent efforts have focussed on simulating the entirety of the procedure to strive towards more substantive high-fidelity simulation and developing means of reproducible and objective assessment, however this is associated with profound technical challenges as demonstrated by Zimmerman et al. The authors developed one of the first physical augmented simulators for mitral TEER which housed an arrangement of injection moulded silicone phantoms, derived from patient MDCT and CMR, to replicate relevant anatomical structures (the venous system from femoral vein to superior vena cava, atria, disposable interatrial septum, and mitral valve) along with an elaborate array of cameras, line lasers and UV lights to simulate TOE and fluoroscopic views (foot pedal activated). The simulator was designed to measure key operator performance metrics including procedure time, time to transeptal puncture, transeptal puncture location, position of the Mitraclip in relation to the valve and correct use of fluoroscopy using eye tracking. Although the platform received positive survey feedback from a small cohort of participants and was able to track appropriate use of fluoroscopy, it was limited by difficulties simulating TOE and fluoroscopy, with the former suffering mismatch between texture and colour and the latter being unable generate adequate images once equipment was advanced into the left heart. Additionally, no consideration was given to haptics and realistic tactile feedback within the model [80].

Bertolini et al. fabricated an alternative TEER simulation platform consisting of a stand mounted translucent 3D printed model of the inferior vena cava, atria, left ventricle (LV) with detachable mitral leaflets. The model was exposed for viewing at key points relevant to TEER to enable visualisation of equipment. Consideration was given to creating realistic haptics with the atria and LV fabricated to a shore hardness of 85A using a blend of Stratasys VeroClear (rigid) and Agilus30 (soft) whilst valve leaflets were printed with pure Agilus30. The blend hardness was selected qualitatively by clinicians following systematic behaviour evaluation of various compositions on cylindrical specimens. The investigators were able to perform four defined procedural steps on the static model (preparation, guide catheter insertion, clip positioning and implantation) under fluoroscopic guidance. Important limitations include the absence of TOE guidance which is crucial to procedural success in TEER as well as the static nature of platform which therefore fails to emulate the challenges of deploying repair devices on a beating heart [81]. These aspects of TEER simulation are increasingly recognised and early attempts at devising dynamic physical TEER simulators are now underway such as the proof-of-concept platform demonstrated by Zhu et al., which comprises a 3D printed ‘cardiac skeleton’ of structures relevant to TEER housed within a water tank, with ports for TOE evaluation and insertion of clip equipment. The mitral valve leaflets were fabricated from echo friendly silicone and were driven by a motor which mimicked the cardiac cycle. The authors were apparently able to perform transseptal puncture, clip navigation within the left atrium and leaflet clipping using fluoroscopy, TOE and camera imaging guidance [82].

Important progress has been made towards high fidelity physical simulation in mitral interventions, however work in this area remains in its infancy with several prevailing barriers. There remains a lack of a standardised curriculum for technical skills training within interventional training programs, particularly in the transcatheter realm, thereby stifling simulator development due to lack of clarity regarding training requirements. Variable attention has been given to realistic haptics and feedback within simulators, this is partly due to limitations with materials technologies but also due to the absence of systematic quantitative evaluation of materials against native tissue and a limited understanding of cardiovascular biomechanics. Moreover, platforms thus far have largely offered static simulation therefore arguably not fully simulating the haemodynamic complexities of beating heart mitral interventions. Maintaining and employing simulation platforms for training is costly, time intensive and requires specialist infrastructure, which undoubtedly limits their widespread uptake within training programmes. Finally, there is also a lack of large-scale validation studies for platforms described in the literature.

### Peri-procedural patient education

Cardiac surgery and transcatheter interventions are associated with significant periprocedural anxiety for patients and their families. Anxiety and distress often stem from a lack of understanding of their cardiac condition and the intervention proposed. Moreover, there is evidence to suggest that peri-procedural anxiety has an impact on outcomes in those undergoing cardiac surgery [83, 84].

3D printed models have the potential to transform patient’s understanding of their pathology and proposed interventions and have been shown to help reduce periprocedural distress and anxiety. Conventional methods of counselling patients regarding their diagnosis and proposed interventions are through diagrams, information leaflets and verbal descriptions. Generic 3D anatomical heart models are now widely available and can be used for counselling patients however these are usually not patient or pathology specific. Hung et al. found the use of a 3D printed patient-specific mitral model in addition to standard patient educational materials to positively impact patient understanding of their condition and enhanced satisfaction compared a control group who were counselled using standard educational materials and a generic 3D printed heart model in a recent randomised questionnaire study [85]. As such the incorporation of patient-specific 3D printed mitral replicas may in future play an integral role in shaping the peri-procedural patient experience.

## Limitations and barriers to clinical translation

Despite promising studies and the novelty associated with the application of transformative technologies such as 3D printing, the literature surrounding their use constitutes small studies and case series, often without significant quantitive data validating their efficacy, impact on patients’ outcome and cost-effectiveness. Therefore, the benefit of these interventions remains unclear and requires large scale clinical trials. However arguably, the scope for clinical studies in this arena is currently limited by nascency of the technologies involved and the rapid flux of multiple devices on various stages of the translational pathway. Indeed, there remains the prospect of clinical trial data being rendered obsolete by the sheer pace of multi-dimensional innovation and technological advancement in this arena. Further barriers stem from the absence of an overarching consensus framework guiding the standardisation of technical workflows (e.g. printing modalities and materials) and the application of 3D printing technology in mitral interventions. The labour-intensive nature of 3D printing workflows which require considerable expertise and capital investment also preclude widespread translation particularly in the absence of uniform financing and reimbursement models in an era of strained health budgets.

Clinical trial data could conceivably lay the foundation of governance frameworks for technical workflows, reimbursement, and quality control, ultimately fostering a wider cost-effective, evidence-based approach.

## Future directions

In view of the significant technical challenges and the rapid pace of development in the realm of mitral valve interventions, disruptive technologies such as 3D-CAM are likely to see increasing uptake as adjuncts to procedural planning and training.

Brisk development continues in materials and 3D printer technologies which will likely shift the paradigm closer to the fabrication of biomechanically realistic mitral valve replicas as demonstrated by the early work using metamaterials. The field of bioprinting continues to receive much attention and holds promise in the fabrication of physiologically and morphologically accurate cardiac tissue. Although of revolutionary potential, this area of research remains in its infancy and is some way from clinical translation, with challenges pertaining to maintaining cell viability and spatial control in the fabrication of complex biological structures as well as tissue functionality and the integration of biofabricated structures within native physiology [13, 86, 87].

The heterogeneity and dynamic nature of mitral pathologies poses a formidable challenge to realistic dynamic biomechanical simulation, particularly as simulation of human cardiac haemodynamics currently relies on MCS which offer limited scope for adjusting physiological variables. Technologies such as computational flow dynamics may enable highly accurate simulation of human physiology and their integration with dynamic physical modelling may offer considerable future dividends in mitral modelling [88].

Extended realities technologies harbour early promise in the arena of mitral valve modelling and intervention particularly in surgical training, virtual proctoring, and procedure planning. There is also the tantalising prospect of intraprocedural integration as demonstrated by Chu et al. where augmented reality optimised intraprocedural TOE guidance facilitated safer NeoChord implantation in a porcine model[89]. Mixed realities technologies may also in future replace fluoroscopic guidance for transcatheter interventions and therefore mitigate the need for intraprocedural radiation exposure[90].

## Conclusion

3D printing technologies carry considerable promise in aiding procedure planning, patient selection, operator training and ultimately delivering personalised care in the rapidly expanding domain of mitral valve interventions. They also appear amenable to synergistic integration with other rapidly evolving ‘transformative technologies’ such as extended realities, machine learning and computational modelling, thereby enhancing the potential clinical dividends particularly in the crucial remits of procedural outcomes and patient safety. However, it is important to appreciate the current limitations with this technology, and caution is required in its widespread application to the clinical decision-making process at this stage. Indeed, 3D printing technologies in the context of heart valve disease remain in their infancy with further advancements required, particularly in the realm of materials technologies to achieve high fidelity simulation of valvular tissue properties and biomechanics. Whilst the use of these technologies currently remains sporadic, there is no doubt about their potential to enhance personalised patient care and procedural outcomes in an area of rapidly expanding clinical need.

## Abbreviations

3D: Three-dimensional
3D-CAM: Three dimension computer assisted modelling
CAD: Computer aided design
CMR: Cardiac magnetic resonance imaging
DICOM: Digital imaging and communication in medicine
FDM: Fusion deposition modelling
LV: Left ventricle
LVOTO: Left ventricular outflow tract obstruction
MAC: Mitral annular calcification
MCS: Mock circulatory system
MDCT: Multidetector computerised tomography
MR: Mitral regurgitation
PJ: Polyjet
PLA: Polylactic acid
SLA: Stereolithography
SLS: Selective laser sintering
TAVI: Transcatheter aortic valve implantation
TOE: Tranesophageal echo
TEER: Transcatheter edge-to-edge repair
TMVr: Transcatheter mitral valve repair
TMVR: Transcatheter mitral valve replacement
TPU: Thermoplastic urethane
